# Interpretable Autoencoders Trained on Single Cell Sequencing Data Can Transfer Directly to Data from Unseen Tissues

**DOI:** 10.3390/cells11010085

**Published:** 2021-12-28

**Authors:** Julie Sparholt Walbech, Savvas Kinalis, Ole Winther, Finn Cilius Nielsen, Frederik Otzen Bagger

**Affiliations:** 1 Center for Genomic Medicine, Rigshospitalet, University of Copenhagen, Blegdamsvej 9, 2100 Copenhagen, Denmark; julwa@regionsjaelland.dk (J.S.W.); savaskina@hotmail.com (S.K.); ole.winther@regionh.dk (O.W.); finn.cilius.nielsen@regionh.dk (F.C.N.); 2 Center for Surgical Science, Zealand University Hospital, Lykkebækvej 1, 4600 Koege, Denmark

**Keywords:** autoencoders (AE), single-cell mRNA-sequencing data, transfer learning, deep learning, artificial neural networks

## Abstract

Autoencoders have been used to model single-cell mRNA-sequencing data with the purpose of denoising, visualization, data simulation, and dimensionality reduction. We, and others, have shown that autoencoders can be explainable models and interpreted in terms of biology. Here, we show that such autoencoders can generalize to the extent that they can transfer directly without additional training. In practice, we can extract biological modules, denoise, and classify data correctly from an autoencoder that was trained on a different dataset and with different cells (a foreign model). We deconvoluted the biological signal encoded in the bottleneck layer of scRNA-models using saliency maps and mapped salient features to biological pathways. Biological concepts could be associated with specific nodes and interpreted in relation to biological pathways. Even in this unsupervised framework, with no prior information about cell types or labels, the specific biological pathways deduced from the model were in line with findings in previous research. It was hypothesized that autoencoders could learn and represent meaningful biology; here, we show with a systematic experiment that this is true and even transcends the training data. This means that carefully trained autoencoders can be used to assist the interpretation of new unseen data.

## 1. Introduction

Deep learning models have been described as the Swiss Army knife of single-cell mRNA analysis [1] and used for denoising [2,3], data simulation [4], imputation of missing values [5,6,7], visualization [8,9,10,11], and decomposition [12,13]. All these applications have proven that this class of models correctly encapsulates the relevant information in a dataset, even from the sparse and often heterogeneous single-cell data. We showed that with specialized training, it is possible to make such models interpretable [14], and more recently, studies from several other groups have been aiming at the interpretation of autoencoder models trained on single-cell data [15,16,17,18,19].

In our implementation [14], we aimed specifically to increase the autoencoders ability to capture interpretable biological features in the bottleneck layer by enforcing a soft orthogonality constraint to prevent entanglement and task-sharing between units [20,21], using the following constraint on the loss:

Loss = mean(x − y ∗ log(x + ε)) + λ ∗ L2_norm(I − WWT)

where x is the input, y is the reconstructed input; y = decode(encode(x)), ε is 1 × 10^−8^ constant, λ is a hyperparameter that determines the impact of the soft orthogonality constraint, W is the weight matrix of the final encoding layer, W^T^ (the transpose matrix) of W, and I-WWT is the orthogonality constraint. This, in essence, penalizes the sharing of tasks over units in the hidden layer during the training process.

Each unit in the bottleneck layer was subsequently interrogated using saliency maps [22,23]. This made it possible to directly map the relationships between input and output of a neural network model and thereby deconvolute the model to highlight salient features (genes) that are activated for a given cell.

Here we investigate the transferability of the model, which is still an open question for further implementation of this type of model and a prerequisite for transfer learning between datasets. We further use an automated hyperparameter optimization step to pursue a more holistic model and to evaluate the use of additional regularization. We found that adding complexity to the model did not improve the performance. We also found the structure of the data in the latent space to match the structure in the input space, even when the model was trained on data from another tissue. This suggests that our model system was able to generalize and capture fundamental biological processes and that this encoding was transferable across distant tissue types such as muscle and mammary gland tissues. By applying saliency maps, we interpreted the salient features in a systematic manner using gene set enrichment analysis (GSEA) [24]. With batch correction techniques, we could further extend generalization across 10x genomics and SMART-seq2 sequencing platforms.

## 2. Materials and Methods

In this study, we used data from Tabular Muris Compendium [25], where data from 12 organs and tissues were included comprising data generated using both 10x genomics and SMART-seq2 (see Table 1 and Supplementary File S1: Section A) for data counts.

Single-cell mRNA datasets were standardized to the logarithm of counts per million log(CPM+1). Datasets were used independently or combined with Seurat batch correction [26]. The combined dataset takes a name from the Smart-seq2 tissue component (Table 1). The autoencoder was implemented in Python v.3.6.8 (https://www.python.org/, accessed on 9 June 2020) using the PyTorch v.1.3.0 deep learning framework (https://pytorch.org/, accessed on 9 June 2020). The neural network was fully connected with 2 layers for the encoding and 2 layers for the decoding process, with a width defined by the number of genes of the dataset (Table 1). Weights were initialized using the Xavier normal distributed initialization [27], and the autoencoder was trained unsupervised with Stocastic gradient descent (SGD) with a fixed Nesterov momentum of 0.9 using a negative log-likelihood (NLL) Poisson loss function and a soft plus the activation function. The soft orthogonality constraint was applied to the bottleneck layer, as described in the introduction, and the autoencoder was trained using early stopping with a hyperparameter optimization, based on [28]. In order to reduce training time, only genes present in the 50 Mus Musculus Hallmark pathways from MSigDB [29] were used in the training process. Underlying patterns captured in the autoencoder were interpreted using Guided backpropagation [23], and the bottleneck layer was visualized using saliency maps. See Supplementary Materials and Methods for further details on the model, deconvolution, and interpretation of saliency maps from guided backpropagation.

The explainability of the model was assessed with GSEA on the saliency maps based on the implementation from [30]. The performance of the models was visually assessed with UMAP [31] and quantitatively predicting cell type with kNN [32], with k = {5...25}. One dataset was used to train the kNN, and multiple datasets were used for testing (see Supplementary S1.5) to quantify the performance using the following measure:

Accuracy = number of correct predictions/(number of correct predictions + number of false predictions)

## 3. Results

**Figure 1:**
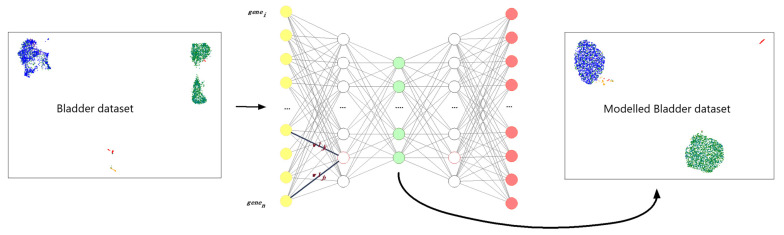
Overview of data visualization and autoencoder. UMAP of raw data (left), autoencoder architecture (center), UMAP of encoded data (right). In the autoencoder nodes in the input layer are yellow, the bottleneck layer nodes are green and output layer nodes are red. “…” indicates additional nodes not displayed in the graphics.

The autoencoder (for example, Bladder data from 10 xgenomics in Figure 1) consists of an input (yellow), output (red), and three hidden layers, the central being the bottleneck layer (green) and thus the lowest dimensionality representation of the data. The input and output dimensions are equal to the number of genes in the dataset that was present in the Mus Musculus Hallmark pathways post-filtering. Initially, the input is encoded into a reduced version in the bottleneck layer and then decoded back to its original dimensionality in the output layer. During the decoding, the autoencoder learns to recreate its original input based on the information retained in the bottleneck layer. The encoding/decoding process is learned during training in a process where weights are fitted based on a Poisson loss function and a soft orthogonality constraint, such that the output values resemble closer the input values, see further specification in Supplementary S1.1).

In order to optimize the model’s hyperparameter space, Bayesian optimization (BO) was used for each individual Tabula Muris tissue dataset, including Smart-seq2, 10x genomics, and combined (see Table 1) (see Supplementary S1.2). The Bayesian statistical model was created by testing some initial hyperparameter values in the actual model and finding the respective loss. The obtained coordinates then constitute the initial Bayesian statistical model [33], and the acquisition function expected improvement (EI) [34] (see below) was used to decide which hyperparameter values should be tested next by using previous experience to avoid optimizing hyperparameters that do not influence the objective function [34,35]. EI(x) = E[max(f(x) − f^∗^),0)] (f is the expected improvements relative to $f^{*}$, $f^{*}=max_{i}y_{i\;}$ is currently the best-observed outcome, with the goal of maximizing f) [33].

An experiment was carried out to investigate a simple model versus a model with additional regularization in the form of L1 norm and weight decay. The simple model was optimized for learning rate (lr), orthogonality constraint, size of the bottleneck layer (bottleneck_size), and the size of the neighboring hidden layers (hidden_size) in the following ranges: lr [1 × 10^−5^, 1 × 10^−3^], orthogonality [1 × 10^−25^, 1 × 10^−5^], hidden_size (50, 140) and bottleneck_size (35, 75). The complex model was additionally optimized for L1 norm and weight decay, in ranges: L1 [1 × 10^−25^, 1 × 10^−5^], weight decay [1 × 10^−25^, 1 × 10^−5^]. The simple and the complex model were optimized over 40 trials, where each trial examined a new set of hyperparameters. Each trial comprised 100 training epochs, where the training dataset constituted 95%. After each trial, a hold-out dataset (5%) evaluated the model’s performance by computing the mean test loss for all cells in the test set. This experiment was performed for Smart-seq2 (Supplementary File S1: Section B) and 10x genomics (Supplementary File S1: Section B), and the combined dataset batch was corrected using Seurat (Table 2; see Supplementary File S1: Section D). Furthermore, we captured the mean loss of unseen datasets evaluated in a model after completed training (see Supplementary File S1: Section E).

We found no clear signs of improvement or deterioration of performance by adding more complexity or when assessing similar experiments for 10x Genomics and SMART-seq2 datasets independently (data not shown). No overall trends point to one model being superior to the other; therefore, further analysis was performed based on the simple model since there is no justification in adding additional complexity, considering the number of extra calculations and computational power needed.

The orthogonality constraint varied markedly between different datasets in 10x genomics, Smart-seq, and Seurat, where optimal values were found in the full range from [1 × 10^−25^, 1 × 10^−5^]. BO always led to a bottleneck layer smaller than the neighboring hidden layers for all datasets and platforms, although the ranges include overlapping values of the hidden_size and the bottleneck_size (50, 140 and 35, 75).

The hyperparameter optimization was able to find minimums in the hyperparameter space to reduce the model’s test loss and thereby enhance the bottleneck layer’s ability to capture essential features in the noisy scRNA-seq dataset. The resulting optimal model and its associated hyperparameters were then trained for additional 400 epochs to constitute the final model.

**Figure 2:**
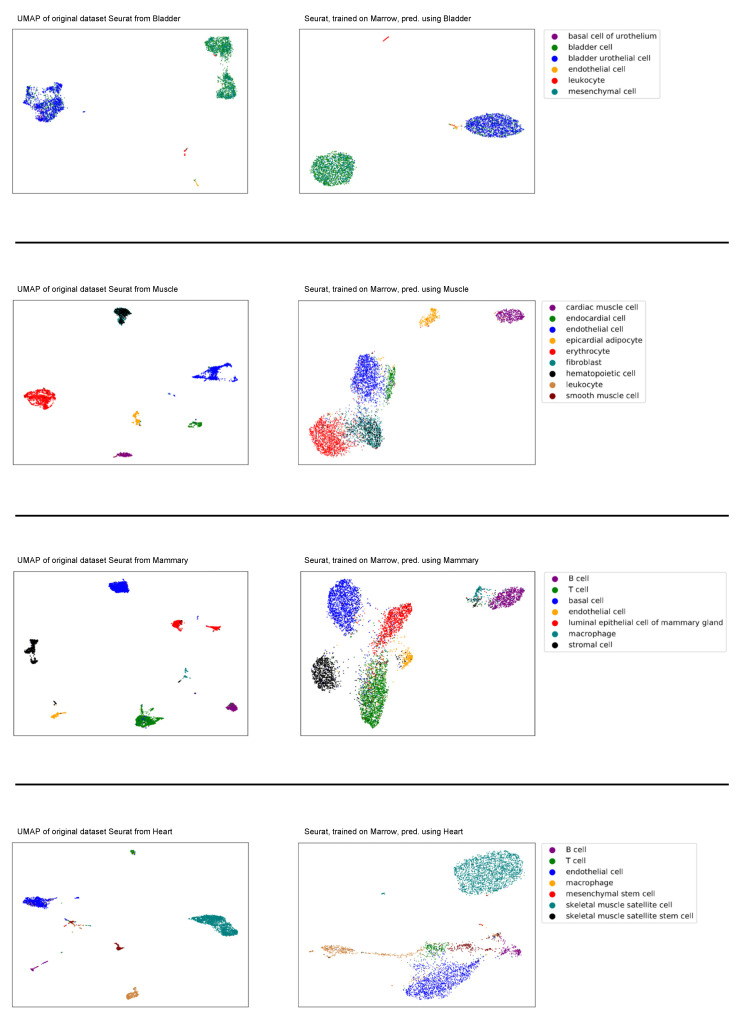
Left plots are the original UMAP of the Seurat datasets, Bladder, Heart, Mammary, Muscle. The right plots are the same datasets e ncoded by the model trained on the Seurat Marrow dataset. Each color depicts a different cell type, as labeled in Tabular Muris Compendium [25].

In order to obtain a better understanding of the underlying space that an autoencoder captures, an autoencoder trained on one dataset was exposed to a new unseen dataset to examine if the model could generalize over datasets. This was performed by feeding a new dataset through an already trained model and examining how the model projects new datasets. For this experiment, we trained the autoencoder with the combined Marrow dataset (Smart-seq2 and 10x genomics) and investigated how well it could project the other combined datasets, see Figure 2. Additionally, we specifically investigated if a model can generalize across other datasets when there are no overlapping cell types, see Supplementary File S1: Section F.

Furthermore, an experiment was conducted assessing one dataset’s ability to predict cell type labels in another dataset. This was performed using the SMART-seq2 Marrow dataset and the combined (Smart-seq2 and 10x genomics) Lung dataset because these represent both integrated and single technology and have an overlap with cells in other tissue datasets. See Table 3 for overlapping cell types with Marrow and Table 4 for overlap with Lung. Cell type distributions for the Smart-seq2 and the combined dataset are seen in Supplementary File S1: Sections C.1 and C.2.

**Figure 3:**
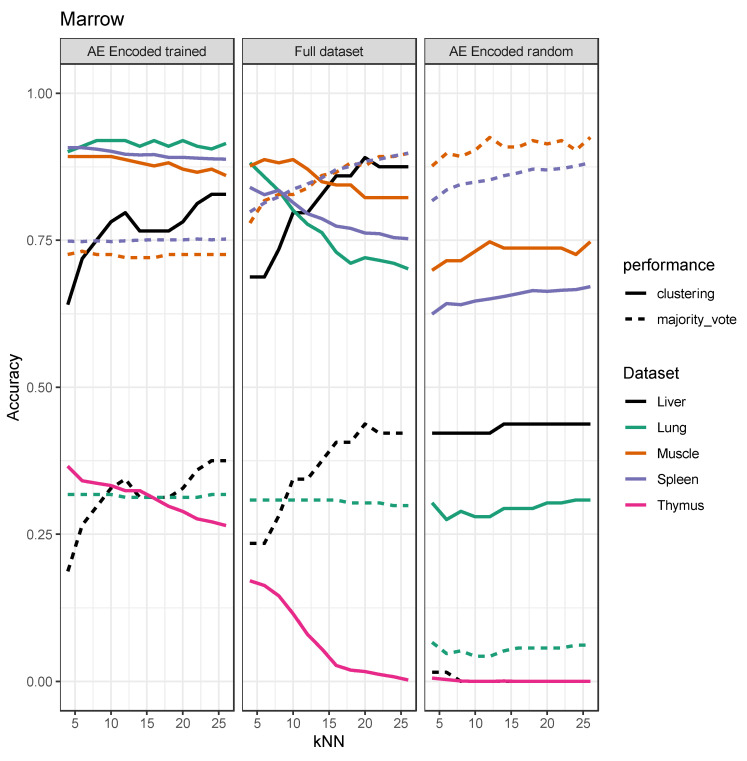
Example kNN-model predicting accuracy of common cell type labels. The kNN was trained on Marrow dataset from Smart-seq2, on the datasets encoded by a trained autoencoder, full dataset, and a randomly initialized autoencoder. Predicting cell type labels for the other Smart-seq2 datasets. Dashed line indicates performance when taking a majority vote as prediction, which assigns all cells to the most abundant cell type. The left represents the kNN model trained and tested on the encoded datasets, the middle represents the kNN model trained and tested on the full dataset and the right represents the kNN trained and tested on data encoded by a randomly initialized autoencoder.

Assessing the kNN predicted cell type, using k = [2, ..., 25] (Figure 3) shows that the bottleneck layer in several cases improves the accuracy of the classification, most likely by acting to denoise the input data, even if the autoencoder is trained on the basis of another dataset. Classifying cell types with a naïve kNN on the original full dataset (middle panel) is thus comparable or worse than the same classification based on autoencoder encoded data even when it is trained on foreign data (left panel). This is not the case when the autoencoder is not trained on any data (right panel). This suggests that relevant information, not specific to the dataset, is encoded in the autoencoder and that it can be transferred to unseen data. This ability may even transfer across not too distant species (see a test in Supplementary File S1: Section I, transferring between Mouse and Human).

**Figure 4:**
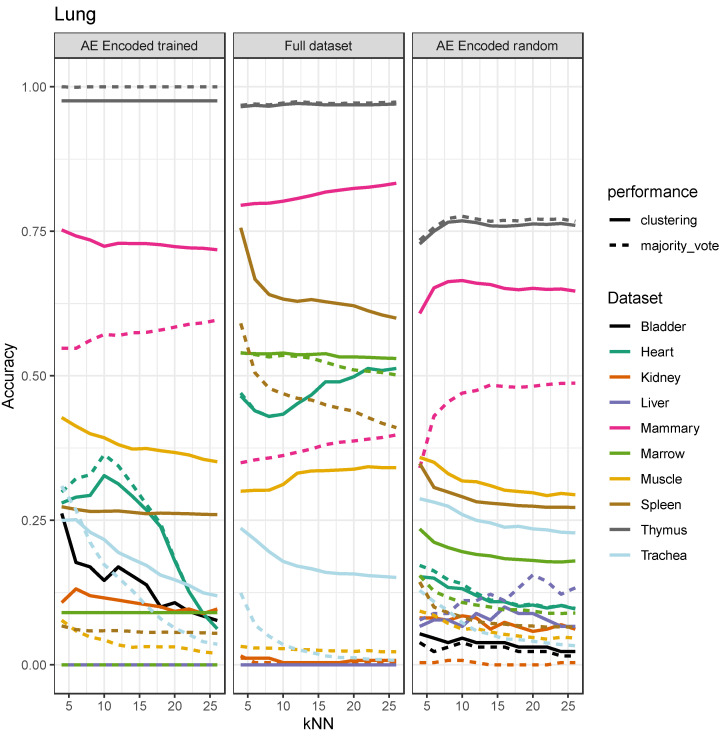
kNN was trained on combined Lung dataset from Smart-seq2 and 10x genomics, on data encoded by the trained autoencoder, full dataset, and dataset encoded by a randomly initialized autoencoder. Predicting cell type labels for the other combined datasets. Dashed line indicates performance when taking a majority vote as prediction, which assigns all cells to the most abundant cell type. The left represents the kNN model trained and tested on the encoded datasets, the middle represents the kNN model trained and tested on the full dataset, and the right represents the kNN trained and tested on data encoded by a randomly initialized autoencoder.

A few of the datasets benefit from denoising, as also seen in Figure 4, but generally, the increase in accuracy is smaller than with the single-technology transfer in Figure 3. Moreover, the effect of neighborhood size, k, for kNN on variance in accuracy appears to be decreased dramatically, most likely by Seurat batch correction. In both cases, the accuracy of predictions using representations from an autoencoder trained on a foreign dataset is comparable to the kNN model trained on the full dataset. This indicates that the autoencoder preserves and encodes essential general biological information in the manifold dimensionality reduction.

There is no assumption that cell types behave identically or are in a similar state when found residing in different tissues or organs, and we merely highlight this overlap to reason that the space spanned by the training space will also accommodate the projected data. When projecting datasets with cell types that the model has not previously been exposed to (Figure 2), the model trained on the Marrow dataset is able to project highly specific heart cells (Supplementary File S1: Section C Table S24). The mean F1 scores corresponding to Figure 3 and Figure 4 can be found in Supplementary File S1: Section G.

We have previously shown that the combination of orthogonality constraint and saliency maps makes it possible to explain features. In order to investigate if this ability is intact when using a foreign autoencoder trained on a different dataset and tissue, we performed GSEA on the input using the gradient of the back-propagated saliencies.

GSEA takes a ranked list of genes as input and investigates the hypothesis that members of a query gene set are randomly distributed throughout the gene set being tested. In this case, the ranking is based on each row in a gene-based saliency map, such that the adapted gradient from the backpropagation pass forms the basis for the ranking.

**Figure 5:**
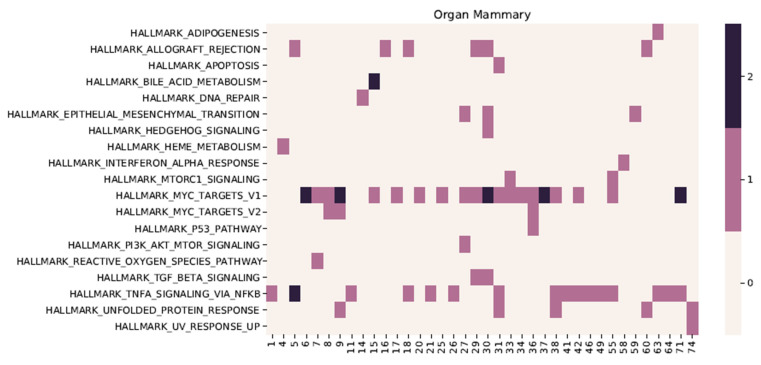
Model trained on Muscle dataset from Smart-seq2 with orthogonality constraint used to compute the saliency maps for each cell type in the Mammary dataset. Heatmap shows frequency that each pathway was significant. The saliency map from each bottleneck layer was thereby used as an input to the GSEA analysis to compute the number of times a given pathway was significant. The *x*-axis represents the (nth-bottleneck layer), and the color of the heatmap displays how many times the given Hallmark pathway was found significant, considering that the mammary dataset has 4 cell types. Only hidden units with at least one significant pathway are displayed.

In order to demonstrate an example of the interpretation of a transferability of the model, it trained on the Muscle dataset from Smart-seq2 that was used to compute the saliency maps for the Mammary dataset (Figure 5). Here the saliency map is computed for each individual cell type in the Mammary dataset and then subtracting the saliency map from the whole dataset. Each individual corrected saliency map was then used as an input to the GSEA analysis to compute the number of times a given pathway was significant.

From the heatmap Figure 5, we can see that hidden units generally do not appear to share tasks when normalized to the total dataset, suggesting intact explainability of the model, as investigated in a previous study [14]. Most of the hidden units have one or few significant pathways associated with their activity, as calculated by the saliency maps. For 19 of the hidden units, only one pathway is significant (11 units have two significant pathways, 6 have 3, and 2 have 4 and 5 pathways, respectively). For the pathways, 11 out of 50 are significant in only one hidden unit, and four have more than four. Out of 50 hallmark signatures that become significant when calculating saliencies for all cells in the Mammary dataset, there are 19 of which two (adipogenesis, epithelial-mesenchymal transition) are also the most significantly overlapping (*p* < 2.27 × 10^−3^, hypergeometric test) with breast tissue-specific genes from Protein Atlas [36]. Signature Hallmark pathways “Myogenesis” and “UV-response UP” that are significantly overlapping with tissue specifically expressed genes from both skeletal muscle and breast tissue are not found significant. These results suggest that the primary signal when interpreting saliencies from the direct transfer of foreign models provides mainly differences between the dataset used for training and the data subsequently passed. Further studies are needed to explore if this feature can be scaled to larger or combined datasets, e.g., to delineate differences in normal cells from cancer cells. In order to further investigate the tendency to share tasks over hidden units, the significant pathways were calculated on a model trained without orthogonality constraint where the remaining parameters are tuned with Bayesian Optimisation (see Supplementary File S1: Section H). Here, only 13 of the hidden are significantly associated with a single pathway (10 units have 2 significant pathways, 6 have 3, and 1 has 5), suggesting that the orthogonality constraint is a contributing factor to ensure interpretability.

## 4. Discussion

Here we investigated the generalization of autoencoders across datasets and found that specialized training of autoencoders encodes biologically meaningful modules, which can be applied to a new dataset. This ability is intact even if there are no cell types in common between training and prediction data. We assume that the primary source of increase in accuracy when predicting cells on the representation of an autoencoder trained from another dataset is due to a reduction in noise. However, the increase would not be possible if the autoencoder was not able to learn a representation that spans biologically relevant features, important to differentiate even unseen cell types. Our findings suggest that a common transcriptional representation can be found using single-cell sequencing. The current main obstacles are the effect of batch and technologies, which we could only partially address here, using common single-cell batch correction methods.

## Figures and Tables

**Table 1:** Datasets from Tabular Muris included in the study and the number of genes in each dataset used for training (after extracting the Hallmark genes and filtering or batch correction).

Smart-seq2	Amount of Genes	10x Genomics	Amount of Genes	Combined Dataset Seurat	Amount of Genes
Tongue	3904	Tongue	10,757	Tongue	498
Thymus	3772	Thymus	7808	Thymus	726
Spleen	3708	Spleen	8798	Spleen	848
Marrow	3992	Marrow	10,445	Marrow	771
Liver	3779	Liver	6950	Liver	653
Kidney	3762	Kidney	11,645	Kidney	774
Heart	4130	Heart_and_Aorta	8081	Heart	745
Bladder	3900	Bladder	10,926	Bladder	653
Mammary	3969	Mammary_Gland	11,495	Mammary	829
Lung	3974	Lung	10,666	Lung	738
Trachea	4022	Trachea	10,584	Trachea	822
Muscle	3968	Limb_Muscle	9202	Muscle	834

**Table 2:** Model test loss using a combined dataset with a simple and a more complex regularization. Bold indicates best model.

	Simple Model Mean Loss	Hidden Layers	Bottleneck Layer	Complex Model Mean Loss	Hidden Layers	Bottleneck Layer
Bladder	**0.94610459**	77	44	0.94966865	101	65
Heart	**0.95382822**	99	57	0.95688325	70	40
Kidney	**0.97204543**	118	42	0.97270389	95	65
Liver	0.95906472	106	35	**0.95659369**	124	40
Lung	0.96714902	83	63	**0.96536231**	119	62
Mammary	0.97676671	65	42	**0.97654885**	91	62
Marrow	**0.98160857**	110	53	0.98427087	67	60
Muscle	**0.95753598**	92	72	0.95977772	89	62
Spleen	**0.97818142**	98	68	0.97743446	67	58
Thymus	**0.98370707**	73	63	0.98711485	88	57
Tongue	0.93583191	90	51	**0.93667495**	74	70
Trachea	0.97082079	100	49	**0.96826011**	116	55

Bold indicates best model—it is standard in machine learning community in performance tables.

**Table 3:** Cell types from SMART-seq2 bone marrow samples, also present in other SMART-seq2 datasets.

Dataset	Cell Types Overlapping with Marrow			
Lung	T-cell	B-cell	Natural killer cell	Monocyte
Liver	B-cell	Natural killer cell		
Muscle	T-cell	B-cell		
Thymus	T-cell	B-cell		
Spleen	T-cell	B-cell		

**Table 4:** Cell types from Lung samples integrated from SMART-seq2 and 10x genomics, also present in other combined datasets.

Dataset	Cell Types Overlapping with Lung				
Bladder	Leukocyte	Endothelial cell			
Marrow	Monocyte	Macrophage	Natural killer cell	T-cell	B-cell
Thymus	T-cell	Leukocyte			
Trachea	Stromal cell	Leukocyte	Epithelial cell	Endothelial cell	
Spleen	B-cell	Myeloid cell	Natural killer cell	T-cell	
Kidney	Leukocyte	Macrophage	Endothelial cell		
Liver	B-cell	Leukocyte	Natural killer cell		
Mammary	Endothelial cell	T-cell	B-cell	Macrophage	
Muscle	Endothelial cell	T-cell	B-cell	Macrophage	
Heart	Endothelial cell	Leukocyte			

## Data Availability

Data analyzed in this paper are publicly available from GEO accession number GSE109774. The code is available at https://github.com/s144489/Autoencoder.git (accessed on 16 December 2021).

## Supplementary Materials

The following is available online at https://www.mdpi.com/article/10.3390/cells11010085/s1: Supplementary S1: Supplementary methods, Section A: Dataset specifications, Section B: Experiment testing simple and complex model, Section C: Cell type distributions from kNN, Section D: Batch correction, Section E: Loss of model trained on one dataset while initializing an unseen dataset, Section F: Auto encoder modelling data with no overlapping cell types, Section G: F1 scores of kNN cell type prediction, Section H: Gene set enrichment analysis of saliency maps. Section I: Autoencoders ability to project Human data.

## References

[1] Way G.P., Greene C.S. (2018). Bayesian deep learning for single-cell analysis. Nat. Methods.

[2] Eraslan G., Simon L.M., Mircea M., Mueller N.S., Theis F.J. (2019). Single-cell RNA-seq denoising using a deep count autoencoder. Nat. Commun..

[3] Grønbech C.H., Vording M.F., Timshel P.N., Sønderby C.K., Pers T.H., Winther O. (2020). scVAE: Variational auto-encoders for single-cell gene expression data. Bioinformatics.

[4] Marouf M., Machart P., Magruder D.S.S., Bansal V., Kilian C., Krebs C.F., Bonn S. (2018). Realistic in silico generation and augmentation of single cell RNA-seq data using Generative Adversarial Neural Networks. bioRxiv.

[5] Mattei P.-A., Frellsen J. MIWAE: Deep Generative Modelling and Imputation of Incomplete Data Sets. Proceedings of the 36th International Conference on Machine Learning.

[6] Hou W., Ji Z., Ji H., Hicks S.C. (2010). A systematic evaluation of single-cell RNA-sequencing imputation methods. Genome Biol..

[7] Viñas Torné R., Azevedo T., Gamazon E., Liò P. (2021). Deep learning enables fast and accurate imputation of gene expression across tissues. Front. Genet..

[8] Bica I., Andrés-Terré H., Cvejic A., Liò P. (2020). Unsupervised generative and graph representation learning for modelling cell differentiation. Sci. Rep..

[9] Ding J., Condon A., Shah S.P. (2018). Interpretable dimensionality reduction of single cell transcriptome data with deep generative models. Nat. Commun..

[10] Lopez R., Gayoso A., Yosef N. (2020). Enhancing scientific discoveries in molecular biology with deep generative models. Mol. Syst. Biol..

[11] Lopez R., Regier J., Cole M.B., Jordan M., Yosef N. (2018). Deep generative modeling for single-cell transcriptomics. Nat. Methods.

[12] Menden K., Marouf M., Oller S., Dalmia A., Magruder D.S., Kloiber K., Heutink P., Bonn S. (2020). Deep learning–based cell composition analysis from tissue expression profiles. Sci. Adv..

[13] Torroja C., Sanchez-Cabo F. (2019). Digitaldlsorter: Deep-Learning on scRNA-Seq to Deconvolute Gene Expression Data. Front. Genet..

[14] Kinalis S., Nielsen F.C., Winther O., Bagger F.O. (2019). Deconvolution of autoencoders to learn biological regulatory modules from single cell mRNA sequencing data. BMC Bioinform..

[15] Mao H., Broerman M.J., Benos P.V. Interpretable Factors in scRNA-seq Data with Disentangled Generative Models. Proceedings of the 2020 IEEE 20th International Conference on Bioinformatics and Bioengineering (BIBE).

[16] Lotfollahi M., Naghipourfar M., Theis F.J., Wolf F.A. (2020). Conditional out-of-distribution generation for unpaired data using transfer VAE. Bioinformatics.

[17] Rybakov S., Lotfollahi M., Theis F.J., Alexander Wolf F. (2020). Learning interpretable latent autoencoder representations with annotations of feature sets. bioRxiv.

[18] Svensson V., Gayoso A., Yosef N., Pachter L. (2020). Interpretable factor models of single-cell RNA-seq via variational autoen-coders. Bioinformatics.

[19] Zhang S., Li X., Lin Q., Lin J., Wong K.-C. (2020). Uncovering the key dimensions of high-throughput biomolecular data using deep learning. Nucleic Acids Res..

[20] Wang W., Yang D., Chen F., Pang Y., Huang S., Ge Y. (2019). Clustering with Orthogonal AutoEncoder. IEEE Access.

[21] Bansal N., Chen X., Wang Z. (2018). Can We Gain More from Orthogonality Regularizations in Training Deep CNNs?. arXiv.

[22] Brocki L., Chung N.C. Concept Saliency Maps to Visualize Relevant Features in Deep Generative Models. Proceedings of the 2019 18th IEEE International Conference on Machine Learning and Applications (ICMLA).

[23] Springenberg J.T., Dosovitskiy A., Brox T., Riedmiller M. (2014). Striving for Simplicity: The All Convolutional Net. arXiv.

[24] Subramanian A., Tamayo P., Mootha V.K., Mukherjee S., Ebert B.L., Gillette M.A., Paulovich A., Pomeroy S.L., Golub T.R., Lander E.S. (2005). Gene set enrichment analysis: A knowledge-based approach for interpreting genome-wide expression profiles. Proc. Natl. Acad. Sci. USA.

[25] Tabula Muris C. (2018). Single-cell transcriptomics of 20 mouse organs creates a Tabula Muris. Nature.

[26] Stuart T., Butler A., Hoffman P., Hafemeister C., Papalexi E., Mauck W.M., Hao Y., Stoeckius M., Smibert P., Satija R. (2019). Comprehensive Integration of Single-Cell Data. Cell.

[27] Glorot X., Bengio Y. Understanding the difficulty of training deep feedforward neural networks. Proceedings of the Thirteenth International Conference on Artificial Intelligence and Statistics, JMLR Workshop and Conference Proceedings, Chia Laguna Resort.

[28] Sutskever I., Martens J., Dahl G., Hinton G. On the importance of initialization and momentum in deep learning. Proceedings of the 30th International Conference on Machine Learning, PMLR.

[29] Liberzon A., Birger C., Thorvaldsdóttir H., Ghandi M., Mesirov J.P., Tamayo P. (2015). The Molecular Signatures Database (MSigDB) hallmark gene set collection. Cell Syst..

[30] Korotkevich G., Sukhov V., Budin N., Shpak B., Artyomov M.N., Sergushichev A. (2021). Fast gene set enrichment analysis. bioRxiv.

[31] Becht E., McInnes L., Healy J., Dutertre C.-A., Kwok I.W.H., Ng L.G., Ginhoux F., Newell E.W. (2018). Dimensionality reduction for visualizing single-cell data using UMAP. Nat. Biotechnol..

[32] Pedregosa F., Varoquaux G., Gramfort A., Michel V., Thirion B., Grisel O., Blondel M., Prettenhofer P., Weiss R., Dubourg V. (2011). Scikit-learn: Machine learning in Python. J. Mach. Learn. Res..

[33] Frazier P.I. (2018). Bayesian Optimization Recent. Advances in Optimization and Modeling of Contemporary Problems.

[34] Wilson J.T., Moriconi R., Hutter F., Deisenroth M.P. (2017). The reparameterization trick for acquisition functions. arXiv.

[35] Letham B., Karrer B., Ottoni G., Bakshy E. (2019). Constrained Bayesian Optimization with Noisy Experiments. Bayesian Anal..

[36] Uhlen M., Oksvold P., Fagerberg L., Lundberg E., Jonasson K., Forsberg M., Zwahlen M., Kampf C., Wester K., Hober S. (2010). Towards a knowledge-based Human Protein Atlas. Nat. Biotechnol..

