# Supplementary material for: Interpretable Autoencoders Trained on Single Cell Sequencing Data Can Transfer Directly to Data from Unseen Tissues
Source: Cells. 2021 Dec 28;11(1):85. doi: 10.3390/cells11010085 (PMC8750521; doi:10.3390/cells11010085)
Supplement: Supplementary file 1 [file cells-11-00085-s001.zip › cells-1459925-supplementary.pdf]

S1 Supplementary methods

S1.1 Model specifications

The morphology of the autoencoder is highlighted in the following, along with the technical specifications and reasoning behind these specifications. The autoencoder was implemented in Python v.3.6.8 using the PyTorch v.1.3.0 deep learning framework.

S1.1.1 Morphology autoencoder

The autoencoder is a fully connected autoencoder, constituted by an input, output and three hidden layers. The bottleneck layer has the lowest dimension of the hidden layers, and the two neighbouring layers have a higher but equal dimension.

S1.1.2 Loss function and orthogonality contraint

The autoencoder was trained using a negative log-likelihood (NLL) Poisson loss function, which is widely used on count data [1], [2]. The loss functions is from the PyTorch library and was used with the parameter *log_input* = *True*, due to the log normalization. The simple loss function is highlighted in the following equation, where the target value refers to the original input and the predicted value refers to the value estimated by the autoencoder, see equation S1.

$$loss = exp(predicted) - target \cdot predicted \; (S1)$$

Loss function with orthogonality constraint

The model was trained using a soft orthogonality, imposed on the bottleneck layer. λ is the hyperparameter that determines the amount of orthogonality added. Equation (S2) is the L2 norm of the orthogonality constraint.

$$\lambda ||W^T W - I||_L^2 \; (S2)$$

W is the weight matrix of bottleneck layer, W^T the transpose matrix of W. I is the identity matrix of W [3], [4]. The orthogonality constraint was added to the loss function since it shows encouraging improvement when training an autoencoder [3], [5]. The full loss function is therefore represented by the NLL Poisson loss function and the orthogonality constraint, see equation (S 3).

$$loss = exp(predicted) - target \times predicted \; + \; \lambda ||W^T W - I||_L^2 (S3)$$

S1.1.3 Weight initialization

The weights was initialized using a normal Xavier initializer, with the addition of a small bias [6]

S1.1.4 Stochastic gradient decent

The autoencoder was trained with Stochastic gradient decent (SGD) with Nesterov momentum (0.9) [7]. SGD is well suited for large data sets [8] and combined with momentum should allow finding the gradient that minimizes the loss function faster than other methods [7].

S1.1.5 Softplus activation function

The Softplus activation function has previously shown to be useful in relation to orthogonal autoencoders modelling single-cell data [2], and was used throughout this project.

$$Softplus(x_{\mathrm{j}}) = ln(1 + e^{input_i})$$

S1.1.6 Regularization

Different regularization method was utilized to avoid overfitting and to enhance the models ability to capture essential features in a large scRNA-seq data.

Weight decay

Weight decay, is a way to penalize the weights of a model [9]. In this case weight decay, was utilized in the hyperparameter optimization, based on previous method [7].

L1 norm

The L1 norm, was used during the hyperparameter optimization. L1 regularization encourages more sparse coefficients by computing the L1 norm of the weights [9]

$$L_1\ regularization = ||W||_L^1$$

Early stopping

Early stopping was implemented to assure that the best observed model constitutes the final model used for further downstream analysis [9].

S1.2 Hyper parameters optimization

This project made use of an automated hyperparameter optimization technique, to avoid manual exploration of the hyperparameter space and simultaneously create a more hollistic model. The hyperparameter optimization, was performed with Bayesian optimization (BO), implemented using the Ax platform v.0.1.6, which is highlighted as a popular framework [10]. Ax is developed by Facebook and is an open source framework.

S1.3 Saliency maps

Traditional gradient based Saliency maps was prone to noise. This let to the development of a variety of adapted methods one of them being Guided backpropagation [11]. Guided backpropagation was used, due to its cleaner visualization [12], additionally *Kinalis et al. 2019* showed that Guided backpropagation can be applied to an autoencoder [2]

Guided backpropagation consist of computing adapted gradients, where all negative gradients are set to zero. This was done for each node of the bottleneck layer, one by one, by assuming all the gradients in the node being tested is equal to one and all other gradients being zero. This method was developed by *Springenberg et al. 2015* [12].

Table S1: Structure of features in a single node

	Gene 1	Gene 2	Gene 3	Gene 4
Cell 1	$\nabla_{1,1}$	$\nabla_{1,2}$	$\nabla_{1,3}$	$\nabla_{1,4}$
Cell 2	$\nabla_{2,1}$	$\nabla_{2,2}$	$\nabla_{2,3}$	$\nabla_{2,4}$
Cell 3	$\nabla_{3,1}$	$\nabla_{3,2}$	$\nabla_{3,3}$	$\nabla_{3,4}$
Cell 4	$\nabla_{4,1}$	$\nabla_{4,2}$	$\nabla_{4,3}$	$\nabla_{4,4}$
Cell 5	$\nabla_{5,1}$	$\nabla_{5,2}$	$\nabla_{5,3}$	$\nabla_{5,4}$
Cell 6	$\nabla_{6,1}$	$\nabla_{6,2}$	$\nabla_{6,3}$	$\nabla_{6,4}$

After computing the adapted gradients for all nodes in the bottleneck layer, there are two methods to proceed with: 1) Extracting information on gene level and 2) extracting information on *Hallmark* pathway level 2). Extracting information of gene-level was done by finding the mean value of the gradients of each gene (mean of the columns in table S1). The gradients matrix in table S1 represent one node in the bottleneck layer, and the mean computation was done for all of these gene matrices. The final Saliency map thereby contains information on the average gradient values for all genes, for each node in the bottleneck layer, see table S2. If extracting information on *Hallmark* pathway level [13], the median was taken for all genes in the *Hallmark* pathway (median of the columns in table S1 present in a given *Hallmark* pathway. Followed by the mean of the median values. This computation was similarly done for all nodes in the bottleneck layer which jointly constitute the Saliency map represented in figure S2.

Table S2: Representation of Saliency map

	Gene 1	Gene 2	Gene 3	Gene 4	Gene 5	Gene 6
Node 1	$\nabla_{1,1}$	$\nabla_{1,2}$	$\nabla_{1,3}$	$\nabla_{1,4}$	$\nabla_{1,5}$	$\nabla_{1,6}$
Node 2	$\nabla_{2,1}$	$\nabla_{2,2}$	$\nabla_{2,3}$	$\nabla_{2,4}$	$\nabla_{2,5}$	$\nabla_{2,6}$
Node 3	$\nabla_{3,1}$	$\nabla_{3,2}$	$\nabla_{3,3}$	$\nabla_{3,4}$	$\nabla_{3,5}$	$\nabla_{3,6}$
Node 4	$\nabla_{4,1}$	$\nabla_{4,2}$	$\nabla_{4,3}$	$\nabla_{4,4}$	$\nabla_{4,5}$	$\nabla_{4,6}$
Node 5	$\nabla_{5,1}$	$\nabla_{5,2}$	$\nabla_{5,3}$	$\nabla_{5,4}$	$\nabla_{5,5}$	$\nabla_{5,6}$
Node 6	$\nabla_{6,1}$	$\nabla_{6,2}$	$\nabla_{6,3}$	$\nabla_{6,4}$	$\nabla_{6,5}$	$\nabla_{6,6}$

Saliency maps was used to investigate underlying patterns, to achieve a comprehensible understanding of the underlying biological features activated in a model, when exposed to a given dataset.

S1.4 Gene set enrichment analysis

The Gene set enrichment analysis (GSEA) was implemented using the R package *FGSEA Simple*. *FGSEA* is known to be a faster implementation, since the algorithm reuse sampling from different query genesets [14]. It is based on a method developed by *Subramanian et al. 2005* [15]. That is efficient in estimating p-values, for testing a whole collection of genesets, but limited in its accuracy. Due to the fact that estimating P-values less than 10^{-6} can be difficult [14]. *FGSEA* was implemented in R v.3.6.1, with the parameters $minSize = 15$, $maxSize = 2000$, $nperm = 100000$, where resulting pathways was considered significant if padj < 0.05.

S1.5 K nearest neighbourg classifier

To explore the trained autoencoders ability to captures cell type relations in the bottleneck layer, a K nearest neighbour (KNN) classifier was trained. KNN algorithm uses the (K) number of nearest neighbor points, to assigns a cell type. Each cell is connected to its (K) closets or most similar points, and the most present cell type will constitute the predicted cell type [16]. The KNN model was trained on one dataset and used to predict the cell type labels of another dataset. This was done for the original gene expression values (x) and the gene expression values encoded by the autoencoder.

In the first scenario: The original gene expression data was used to train the KNN model. Another dataset was then tested in the KNN model. In the second scenario: The original gene expression data was encoded using the autoencoder (encode(x)) and these encoded values was then used to train the KNN model. Likewise was the tested dataset encoded (encode(x)). In both cases the trained and tested dataset was within the same dataset category (Smart-seq2, Drop-seq, Harmony or Seurat). The dataset tested in the KNN model was filtered for the cell types present in the dataset that trained the KNN model, in order to find the prediction accuracy, using equation (S4).

$$Accuracy = \frac{\text{number of correct prediction}}{(\text{number of correct predictions + number of false predictions})} (S4)$$

This was done using the KNeighborsClassifier from the *scikit-learn*,with default parameters and varied n_neighbors [17].

S1.6 Procedure

- Data loading and data processing

- Training the autoencoder
- Implementation of hyperparameter optimization
- Running new data trough a trained model
  - Imputation of missing values
- Saliency maps
- Gene set enrichment analysis

S1.6.1 Data loading and data processing

First step in running the autoencoder, is loading the data and processing the data. this is seen in figure S1. In step 2) cells were randomly shuffled and the genes with no count value above two was removed. To train on genes with a specific known function the *Hallmark* genes was extracted from the dataset for subsequent training. These *Hallmark* genes is constituted by the genes present in the *Mus Musculus Hallmark* pathways from MSigDB [13]. The Seurat and Harmony datasets had already gone trough normalization during the pre-processing step, therefore only the Smart-seq2 and Drop-seq datasets where normalized using counts per million (CPM).

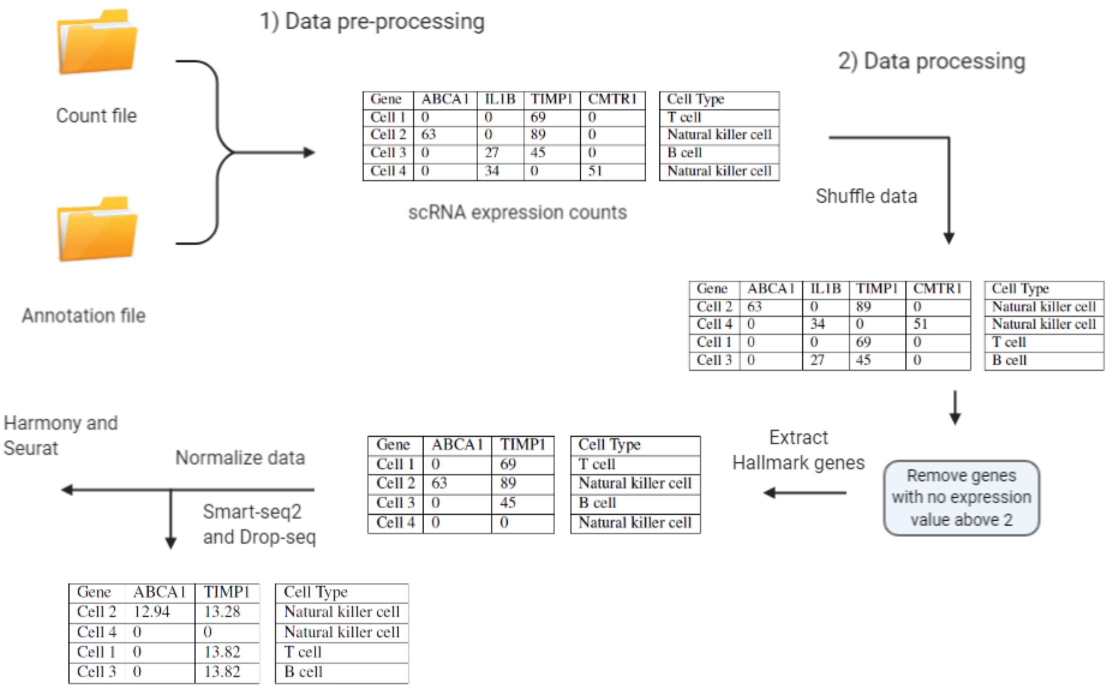

Figure S1: A stepwise description of the data loading process. Step 1) highlights the pre-processing step and step 2) the subsequent loading and processing, prior to training the model.

Thereafter, the data was divided into test data and training data. The training data constituted 95 % of cells, and the test dataset constituted 5 % of the cells in the dataset. The autoencoder was

trained with a batch size of 1, since the aim of the model, is to learn underlying features for each single-cell and not for a batch of cells.

S1.6.2 Training the autoencoder

To capture dependencies in data and possibly derive novel biological information. The autoencoder needs to be trained. This is briefly touched upon in the following.

Training is done is a stepwise process, where each cell in the training data, is feed trough the autoencoder, in the following steps.

- Run cell (x) trough the autoencoder to predict y, y = decode(encode(x))
- Compute the loss between x and y using the loss function (Negative log likelihood Poisson loss function)
- Add regularization to the loss e.g. orthogonality constraint
- Backpropogate trough the network to compute all gradients
- Use these gradient to update the weights in a direction that minimize the loss.
- Do this for all cells in the dataset.

The above described process constitutes the training of 1 epochs. After every 10 epochs the test loss is computed. This is done by running all cells in the test dataset (x_test), trough the autoencoder (y_test = decode(encode(x_test))) and computing the average loss between x_test and y_test, using the NLL Poisson loss function and the added regularization. If the computed test loss decreases, compared to previous lowest test loss, the model was saved. If the test loss between 20 epochs did not change by more than 10^{-8} the training was stopped. Thereby the network completed its training after a total amounts of epochs or if network stopped improving. The optimal model was then re-loaded, for the purpose of clustering the cells using UMAP and PCA and computing Saliency maps. Prior to training the autoencoder, hyperparameter optimization took place, which uses the same concept for training, while simultaneously optimizing hyperparameters.

S1.6.3 Implementation of hyperparameter optimization

Hyperparameter optimization was performed, to minimize the test loss and enhance the change of extracting useful biological information from the bottleneck layer. The hyperparameter optimization was done over 40 trials, where different hyperparameter values was tested in a prior defined range. Each trial trained for 100 epochs and returned the test loss and the associated hyperparameters. After all trials, the best model and associated hyperparameters was reloaded for subsequent training of 400 epochs following the training procedure highlighted in section S1.6.2.

S1.6.4 Running new data trough a trained model

In order to investigate how the trained model was able to model unseen datasets. A dataset within the same dataset category (Smart-seq2, Drop-seq or Seurat) was loaded. In this process the genes from the dataset used for training was intersected with the new dataset, and ordered accordingly. If values were missing for some genes they were imputed.

Imputation of missing values

The imputation of missing gene values consists of finding the percentage of zero values. This is done by computing the minimum values of all genes, in the new dataset and taking the mean of these values. The min.mean() value is then considered the new threshold for "zero value". This threshold is then used to calculate the average percentage of values below the threshold, which is considered "zero values". The estimated percentage of "zero values", for the missing gene, was randomly added by inferring the min.mean() and all other values constituted mean.min() of the new dataset.

This method was chosen since scRNA-seq data contain many zero values, but the batch corrected dataset might not have zero as the most occurring value. Instead the most occurring value can be represented by another number. Therefore this imputation is a more general way to impute missing gene values and simultaneously account for the Poisson distribution.

S1.6.5 Saliency maps

When the autoencoder had completed its training. The optimal model was re-loaded, for the purpose of computing the Saliency maps, for each individual cell type and for the whole dataset, see section S1.3. This was initially done for the dataset used to train the model, but subsequently for a new unseen dataset. Thereby the optimal model of a given dataset was loaded. Then a new dataset was run trough the model and intersected with the dataset used to train the model. Finally the resulting gene-level Saliency maps was used for the GSEA analysis.

S1.6.6 Gene set enrichment analysis

Recall that GSEA takes a pre-ranked geneset as input and investigates if members of query geneset are randomly distributed throughout the geneset being tested or primarily found at the top [15] (refer to section S1.4 for more information on GSEA). In this case each node in the Saliency map constitutes the pre-ranked geneset that was used for GSEA analysis. The pre-ranked geneset corresponds to the gene-level statistics of a node in the Saliency map, this is highlighted in table S3. Where the gene-level statistics, in each node has been converted into z-values (x - mean(x)) / standard deviation(x)).

Table S3: Snip of Saliency map

	Gene 1	Gene 2	Gene 3	Gene 4	Gene 5	Gene 6	Gene 7
Node 1	z1	z1	z1	z1	z1	z1	z1
Node 2	z2	z2	z2	z2	z2	z2	z2
Node 3	z3	z3	z3	z3	z3	z3	z3
Node 4	z4	z4	z4	z4	z4	z4	z4

However a dataset, representing a certain organ or tissue, requires finding a Saliency map for each individual cell type and the whole dataset. The Saliency map for the whole dataset was then substracted all cell type specific Saliency maps, prior to computing the z-values. To account for overall biological signals that the model learned and enhance the chances of pin pointing signals significant for a certain organ, and not just general signals that the model has learned. The corrected cell specific Saliency maps, then constitutes the input to the GSEA analysis. The GSEA analysis compares a pre-ranked geneset from the Saliency map to query pathways in the MSigDB. If a given pre-ranked geneset had a padj < 0.05, the geneset was considered significant if upregulated.

The uniting of GSEA and Saliency maps, was used to explore if specific biological features were activated certain places in the neural network model and to get a better understanding of how biological features is captured in the bottleneck layer.

Section A

Dataset specifications

Table S4: Smart-seq2, Data information after extracting the Hallmark genes and filtering genes that had a sum of expression less than 2

**Tabula Muris Smart-seq2**				
Organ	Amount of Genes	Amount of Cells	Amount of cell types	Percentage of zeroes pr. Cell
Bladder	3900	1287	3	59.49%
Heart	4130	4534	9	78.53%
Kidney	3762	517	6	85.02%
Liver	3779	710	5	70.08%
Lung	3974	1620	16	78.92%
Mammary	3969	2304	4	69.01%
Marrow	3992	4897	8	75.56%
Muscle	3968	1937	7	79.67%
Spleen	3708	1689	3	83.92%
Thymus	3772	1283	2	83.08%
Tongue	3904	1394	2	61.47%
Trachea	4022	846	4	70.71%

Table S5: 10xgenomics, Data information after extracting the Hallmark genes and filtering genes that had a sum of expression less than 2

**Tabula Muris 10xgenomics**				
Organ	Amount of Genes	Amount of Cells	Amount of cell types	Percentage of zeroes pr. Cell
Bladder	10926	3186	4	67.33%
Heart_and_Aorta	8081	2529	4	70.25%
Kidney	11645	3339	8	78.21%
Limb_Muscle	9202	2937	6	81.19%
Liver	6950	2266	4	77.11%
Lung	10666	3117	13	81.28%
Mammary_Gland	11495	3368	7	80.44%
Marrow	10445	2984	14	73.97%
Spleen	8798	2645	5	83.44%
Thymus	7808	2357	3	69.91%
Tongue	7538	10757	3	65.86%
Trachea	10584	3237	5	83.31%

Table S6: Combined 10xgenomics and Smart-seq2,Data information after batch correction and extracting the Hallmark genes and filtering genes that had a sum of expression less than 2

**Combined 10xgenomics and Smart-seq2 data batch corrected using Seurat**			
	Amount of Genes	Amount of Cells	Amount of cell types
Bladder	653	3787	6
Heart	745	5060	9
Kidney	774	3298	13
Liver	653	2555	7
Lung	738	7024	23
Mammary	829	6785	7
Muscle	834	5846	7
Marrow	771	8549	18
Spleen	848	11241	6
Thymus	726	2712	5
Tongue	498	8932	3
Trachea	822	12094	7

Section B

Experiment testing simple and complex model

Table S7: Smart-seq2, Compares the mean test loss of the complex (lr, orthogonality, hidden_size, bottleneck_size, L1, weight decay and the simple model (lr, orthogonality, hidden_size, bottleneck_size)

Smart-seq2		
**Organ**	**Simple model loss**	**Complex model los**
Bladder	0.328127561	-0.67577557
Heart	0.558011589	0.603821717
Kidney	0.594373616	0.698023797
Liver	-0.37875915	-0.592997629
Lung	0.466990837	0.432692507
Mammary	0.087807704	0.075413681
Marrow	0.452254044	0.444665979
Muscle	0.491863511	0.461258427
Spleen	0.663175052	0.666026653
Thymus	0.626973583	0.661040656
Tongue	-0.665483642	-0.539805402
Trachea	-0.167571426	-0.281279303

Table S8: 10xgenomics, Compares the mean test loss of the complex (lr, orthogonality, hidden_size, bottleneck_size, L1, weight decay and the simple model (lr, orthogonality, hidden_size, bottleneck_size)

10xgenomics		
**Organ**	**Simple model loss**	**Complex model los**
Bladder	-0.13859364	-0.178374164
Heart and Aorta	-0.497399108	-0.337941112
Kidney	0.298035188	0.37422153
Limb Muscle	0.524737142	0.507086877
Liver	0.220362172	0.268167226
Lung	0.602095967	0.604511011
Mammary Gland	0.509200782	0.469836471
Marrow	0.219094117	0.121379251
Spleen	0.718774260	0.735692292
Thymus	-0.269887634	-0.310725786
Tongue	0.143107637	0.944072607
Trachea	0.733077296	0.744878366

Section C

Cell type distributions from kNN

Section C.1 Smart-seq2

Table S9: Cell type distribution of the Smart-seq2 Marrow dataset that was used to train the kNN model

Marrow	Frequency	Percentage
B cell	1848	37.74
Hematopoietic stem cell	1291	26.36
Neutrophil	599	12.23
Granulocyte	380	7.76
Monocyte	324	6.62
Fraction A pre-pro B cell	177	3.61
T cell	142	2.90
Natural killer cell	136	2.78
Sum	4897	100.0

Table S10: Cell type distribution of the Smart-seq2 Lung dataset that was tested in the kNN model

**Lung**	**Frequency**	**Percentage**
Endothelial cell	738	45.56
Stromal cell	366	22.59
type II pneumocyte	94	5.80
dendritic cell	69	4.26
Macrophage	69	4.26
Monocyte	65	4.01
B cell	55	3.40
T cell	55	3.40
Natural killer cell	36	2.22
Leukocyte	31	1.91
Ciliated cell	14	0.86
Clara cell	13	0.80
Epithelial cell	9	0.56
Type I pneumocyte	2	0.12
Mesothelial cell	2	0.12
Lung neuroendocrine cell	2	0.12
Sum	1620	100

Table S11: Cell type distribution of the Smart-seq2 Muscle dataset that was tested in the kNN model

**Muscle**	**Frequency**	**Percentage**
Skeletal muscle satellite cell	546	28.19
Mesenchymal stem cell	486	25.09
skeletal muscle satellite stem cell	442	22.82
Endothelial cell	206	10.64
B cell	151	7.80
Macrophage	71	3.67
T cell	35	1.81
Sum	1937	100

Table S12: Cell type distribution of the Smart-seq2 Thymus dataset that was tested in the kNN model

**Thymus**	**Frequency**	**Percentage**
T cell	1250	97.43
Mesenchymal stem cell	33	2.57
Sum	1283	100

Table S13: Cell type distribution of the Smart-seq2 Liver dataset that was tested in the kNN model

**Liver**	**Frequency**	**Percentage**
Hepatocyte	399	56.20
Endothelial cell of hepatic sinusoid	196	27.61
Kupffer cell	51	7.18
Natural killer cell	35	4.93
B cell	29	4.08
Sum	710	100.00

Section C.2 Seurat

Table S14: Cell type distribution of the combined Smart-seq2 and 10xgenomics Lung dataset that was used to train the kNN model with Seurat data

**Lung**	**Frequency**	**Percentage**
Stromal cell	2906	41.37
Natural killer cell	860	12.24
Endothelial cell	738	10.51
Lung endothelial cell	462	6.58
Alveolar macrophage	345	4.91
T cell	302	4.30
B cell	259	3.69
Non-classical monocyte	220	3.13
Leukocyte	183	2.61
Type II pneumocyte	183	2.61
Classical monocyte	161	2.29
Myeloid cell	87	1.24
Macrophage	69	0.98
Dendritic cell	69	0.98
Monocyte	65	0.93
Ciliated columnar cell of tracheobronchial tree	49	0.70
Mast cell	24	0.34
Ciliated cell	14	0.20
Clara cell	13	0.19
Epithelial cell	9	0.13
Lung neuroendocrine cell	2	0.03
Mesothelial cell	2	0.03
Type I pneumocyte	2	0.03
Sum	7024	100

Table S15: Cell type distribution of the combined Smart-seq2 and 10xgenomics Bladder that was tested in the kNN model

**Bladder**	**Frequency**	**Percentage**
Bladder cell	1735	45.81462899
Bladder urothelial cell	1167	30.8159493
Mesenchymal cell	656	17.3224188
Basal cell of urothelium	99	2.614206496
Leukocyte	73	1.927647214
Endothelial cell	57	1.505149195
Sum	3787	100

Table S16: Cell type distribution of the combined Smart-seq2 and 10xgenomics Marrow that was tested in the kNN model

**Marrow**	**Frequency**	**Percentage**
B cell	1848	21.62
hematopoietic stem cell	1291	15.10
Granulocyte	1105	12.93
Monocyte	849	9.93
Neutrophil	599	7.01
hematopoietic precursor cell	392	4.59
granulocytopoietic cell	378	4.42
T cell	304	3.56
late pro-B cell	265	3.10
proerythroblast	265	3.10
promonocyte	257	3.01
Fraction A pre-pro B cell	243	2.84
Macrophage	223	2.61
Erythroblast	155	1.81
Natural killer cell	136	1.59
Immature B cell	113	1.32
Early pro-B cell	65	0.76
Basophil	61	0.71
Sum	8549	100.00

Table S17: Cell type distribution of the combined Smart-seq2 and 10xgenomics Thymus that was tested in the kNN model

Thymus	Frequency	Percentage
immature T cell	1354	49.93
T cell	1250	46.09
DN1 thymic pro-T cell	44	1.62
Mesenchymal stem cell	33	1.22
Leukocyte	31	1.14
Sum	2712	100

Table S18: Cell type distribution of the combined Smart-seq2 and 10xgenomics Trachea that was tested in the kNN model

Trachea	Frequency	Percentage
Mesenchymal cell	7848	64.89
Blood cell	1139	9.42
Endothelial cell	1061	8.77
Epithelial cell	999	8.26
Stromal cell	569	4.70
Neuroendocrine cell	362	2.99
Leukocyte	116	0.96
Sum	12094	100

Table S19: Cell type distribution of the combined Smart-seq2 and 10xgenomics Spleen that was tested in the kNN model

Spleen	Frequency	Percentage
B cell	8124	72.27
T cell	2333	20.75
Macrophage	464	4.13
Natural killer cell	230	2.05
Myeloid cell	48	0.43
Dendritic cell	42	0.37
Sum	11241	100

Table S20: Cell type distribution of the combined Smart-seq2 and 10xgenomics Kidney that was tested in the kNN model

**Kidney**	**Frequency**	**Percentage**
Kidney proximal straight tubule epithelial cell	1198	36.33
Kidney loop of Henle ascending limb epithelial cell	471	14.28
Kidney collecting duct epithelial cell	443	13.43
Kidney capillary endothelial cell	392	11.89
Kidney tubule cell	261	7.91
Macrophage	139	4.21
Leukocyte	80	2.43
Fenestrated cell	69	2.09
Fibroblast	65	1.97
Mesangial cell	51	1.55
Kidney cell	45	1.36
Kidney collecting duct cell	44	1.33
Endothelial cell	40	1.21
Sum	3298	100.00

Table S21: Cell type distribution of the combined Smart-seq2 and 10xgenomics Liver that was tested in the kNN model

**Liver**	**Frequency**	**Percentage**
Hepatocyte	2163	84.66
Endothelial cell of hepatic sinusoid	224	8.77
Kupffer cell	51	2.00
Natural killer cell	35	1.37
B cell	29	1.14
Duct epithelial cell	27	1.06
Leukocyte	26	1.02
Sum	2555	100

Table S22: Cell type distribution of the combined Smart-seq2 and 10xgenomics Mammary that was tested in the kNN model

**Mammary**	**Frequency**	**Percentage**
T cell	1750	25.79
Basal cell	1667	24.57
Stromal cell	1128	16.62
Luminal epithelial cell of mammary gland	1011	14.90
B cell	743	10.95
Endothelial cell	300	4.42
Macrophage	186	2.74
Sum	6785	100

Table S23: Cell type distribution of the combined Smart-seq2 and 10xgenomics Muscle that was tested in the kNN model

**Muscle**	**Frequency**	**Percentage**
Mesenchymal stem cell	1622	27.75
Endothelial cell	1536	26.27
Skeletal muscle satellite cell	900	15.40
B cell	612	10.47
Skeletal muscle satellite stem cell	442	7.56
Macrophage	379	6.48
T cell	355	6.07
Sum	5846	100

Table S24: Cell type distribution of the combined Smart-seq2 and 10xgenomics Heart that was tested in the kNN model

**Heart**	**Frequency**	**Percentage**
Fibroblast	2313	45.71
Endothelial cell	1445	28.56
Leukocyte	488	9.64
Smooth muscle cell	255	5.04
Endocardial cell	238	4.70
Cardiac muscle cell	200	3.95
Epicardial adipocyte	93	1.84
Hematopoietic cell	17	0.34
Erythrocyte	11	0.22
Sum	5060	100.00

Section D
Batch correction

Figure S2 highlighting the combined Marrow datasets from Smart-seq2 and 10Xgenomics after Seurat batch correction.

Figure S2: Combines Smart-seq2 and 10Xgenomics dataset from Marrow after batch correction.

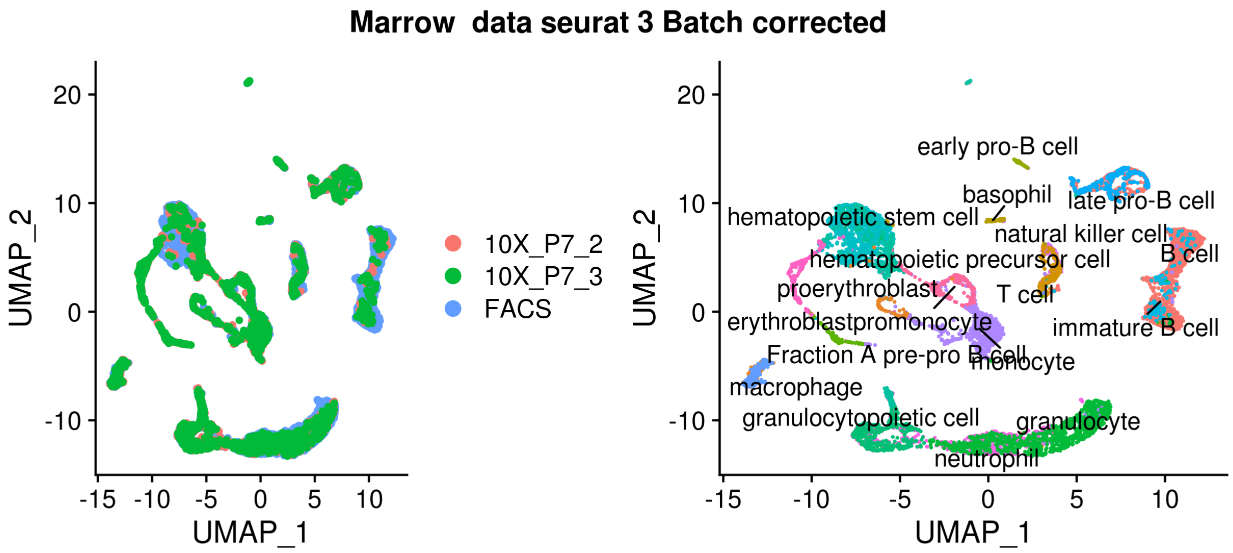

Section E

Loss of model trained on one dataset while initializing an unseen dataset

Table S25: Combined datasets Smart-seq2 and 10XGenomics (Seurat). Table highlighting the loss when passing an entire dataset trough a pretrained model and then computing the mean loss.

**Model used to test loss of other datasets**												
	Bladder	Heart	Kidney	Liver	Lung	Mammary	Marrow	Muscle	Spleen	Thymus	Tongue	Trachea
Bladder	0.962991	0.964969	1.006564	1.010277	0.979975	0.986827	1.003034	1.003608	1.006263	1.005678	1.010527	0.972455
Heart	0.98041	0.963591	0.995004	1.013166	0.993267	0.992322	1.001604	0.974789	1.011841	1.00527	1.002127	0.994327
Kidney	1.009506	0.970997	0.962901	1.02125	1.002956	0.998612	1.002243	0.991229	1.011263	1.00471	1.011639	0.992795
Liver	1.00619	1.001828	1.006839	0.958677	1.003829	0.996457	1.001526	1.005507	0.998042	1.003379	1.009158	1.002372
Lung	0.975633	0.994729	0.998711	1.020311	0.965123	0.986624	1.001779	0.985898	1.005702	1.007295	1.009135	0.991078
Mammary	0.97993	0.965682	1.004285	1.011161	0.973639	0.966046	0.999557	0.997255	1.003229	1.004757	1.008219	1.000525
Marrow	1.008467	1.001427	1.006272	1.012516	1.005536	1.002565	0.978043	0.996591	1.006852	0.992527	1.011814	1.003005
Muscle	1.006434	0.986018	0.985477	1.017745	0.990823	0.993208	1.004094	0.91518	1.001118	1.005435	1.006649	0.957935
Spleen	1.008785	0.996874	1.003569	1.004442	0.992019	0.989158	0.993563	0.990733	0.973677	0.999803	1.005547	0.99954
Thymus	1.006467	1.000429	1.006874	1.012241	1.008001	1.002022	0.997833	1.004976	1.009062	0.989049	1.013673	1.002187
Tongue	1.002108	0.996942	0.99818	1.006087	0.997781	0.995673	1.00052	0.993459	1.005064	1.001221	0.94344	0.997394
Trachea	1.003216	0.997523	1.002763	1.011465	0.981995	0.990636	1.001667	0.996266	1.018151	1.005542	1.009235	0.965983

Section F

Auto encoder modelling data with no overlapping cell types

Below is seen four UMAPs. 1) The original Bladder dataset Smartseq-2. 2) The UMAP of the encoded bladder dataset by a model trained on the Bladder data. 3) Umap of the Bladder dataset, when encoded by a model trained on the Heart dataset.4) The Heart dataset encoded by the model trained on the heart data. This highlight that all though the Heart and Bladder dataset has no overlapping cell types the heart model is still able to learn features related to the cell types in the Bladder dataset.

Figure S3: UMAP of Smart-seq2 Bladder dataset

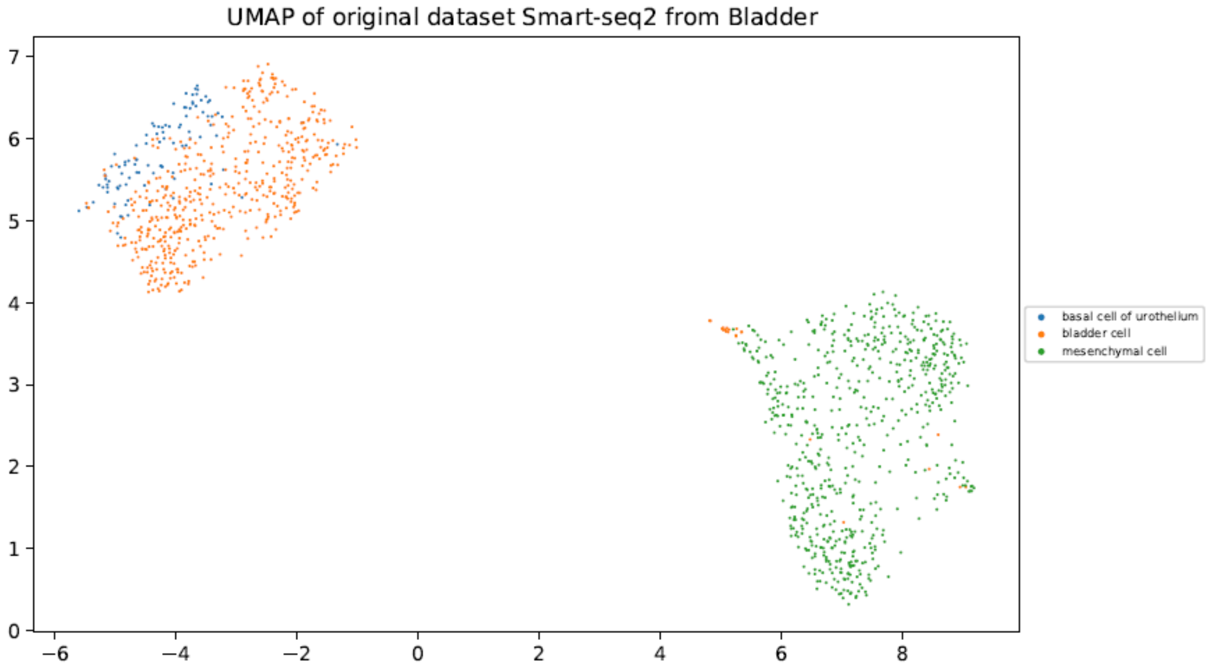

Figure S4: UMAP of Smart-seq2 Bladder dataset encoded by the model trained on the Bladder dataset

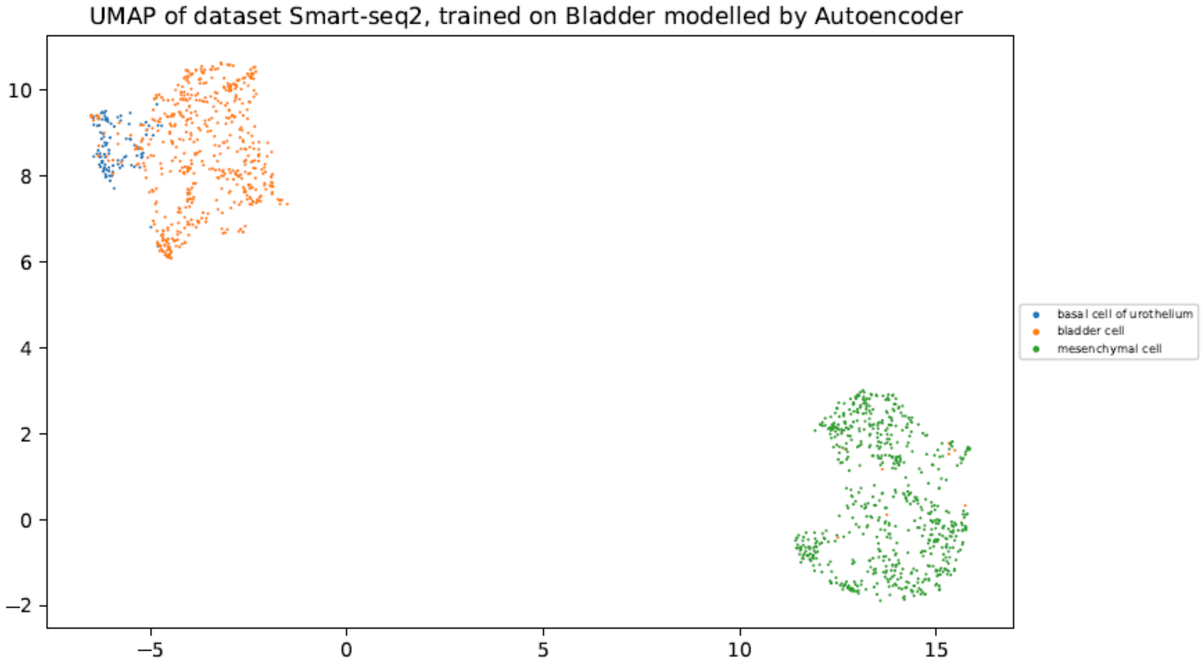

Figure S5: UMAP of Smart-seq2 Bladder dataset encoded by the model trained on the Heart dataset

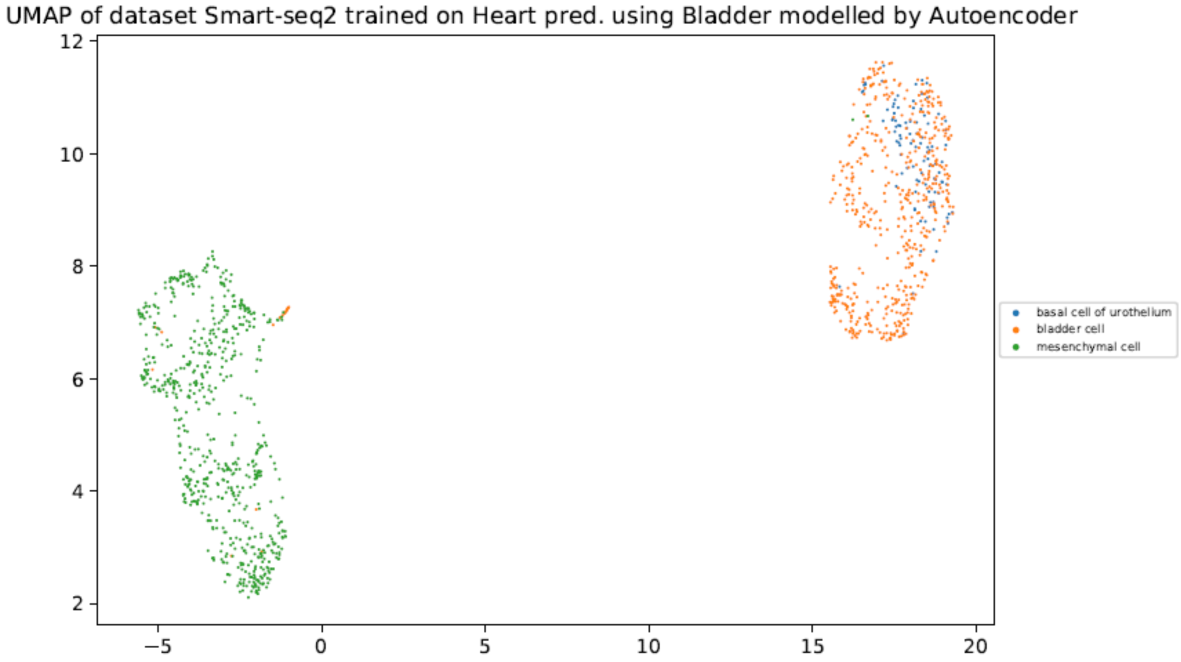

Figure S6: UMAP of Smart-seq2 heart dataset encoded by the model trained on the heart data

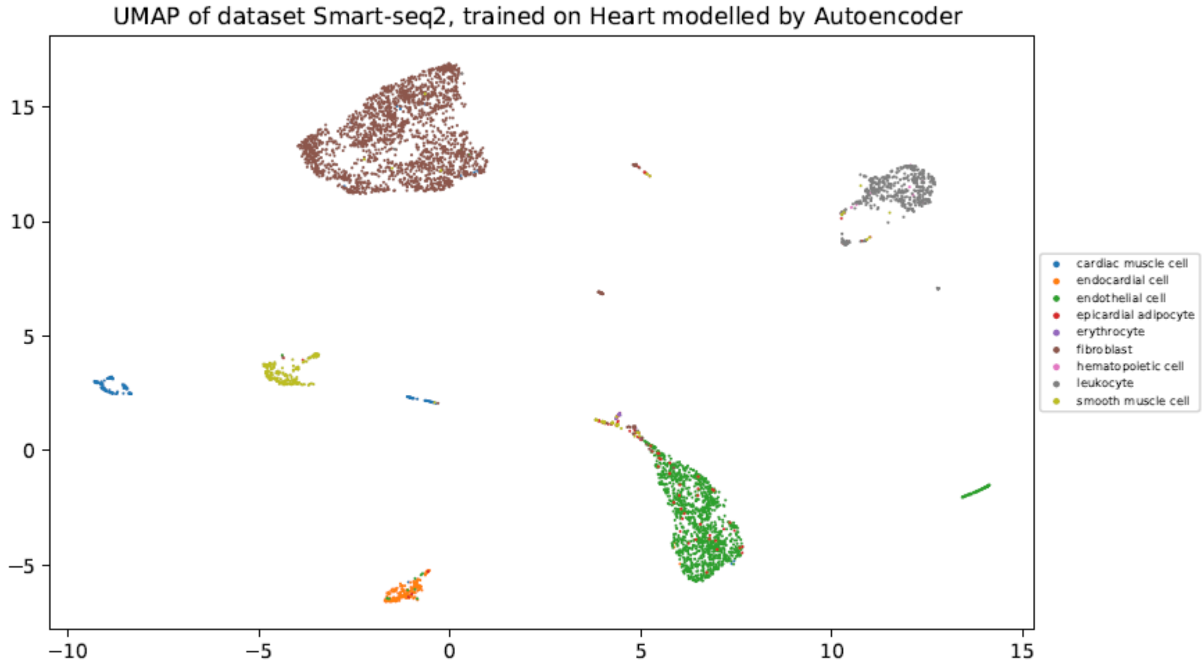

Section G
F1 scores of kNN cell type prediction

After predicting the cell type labels for a new unseen dataset using the kNN model. The mean F1 score across the different cell types was ccalculated for 1), 2) and 3). 1) training the KNN on encoded data based on trained autoencoder, 2) encoded data based on solely an initialized autoencoders random weights (Xavier) and 3) the full dataset.

Figure S7: Mean F1 scores for the cell types in the Lung dataset from combined Smart-seq2 and 10xgenomics

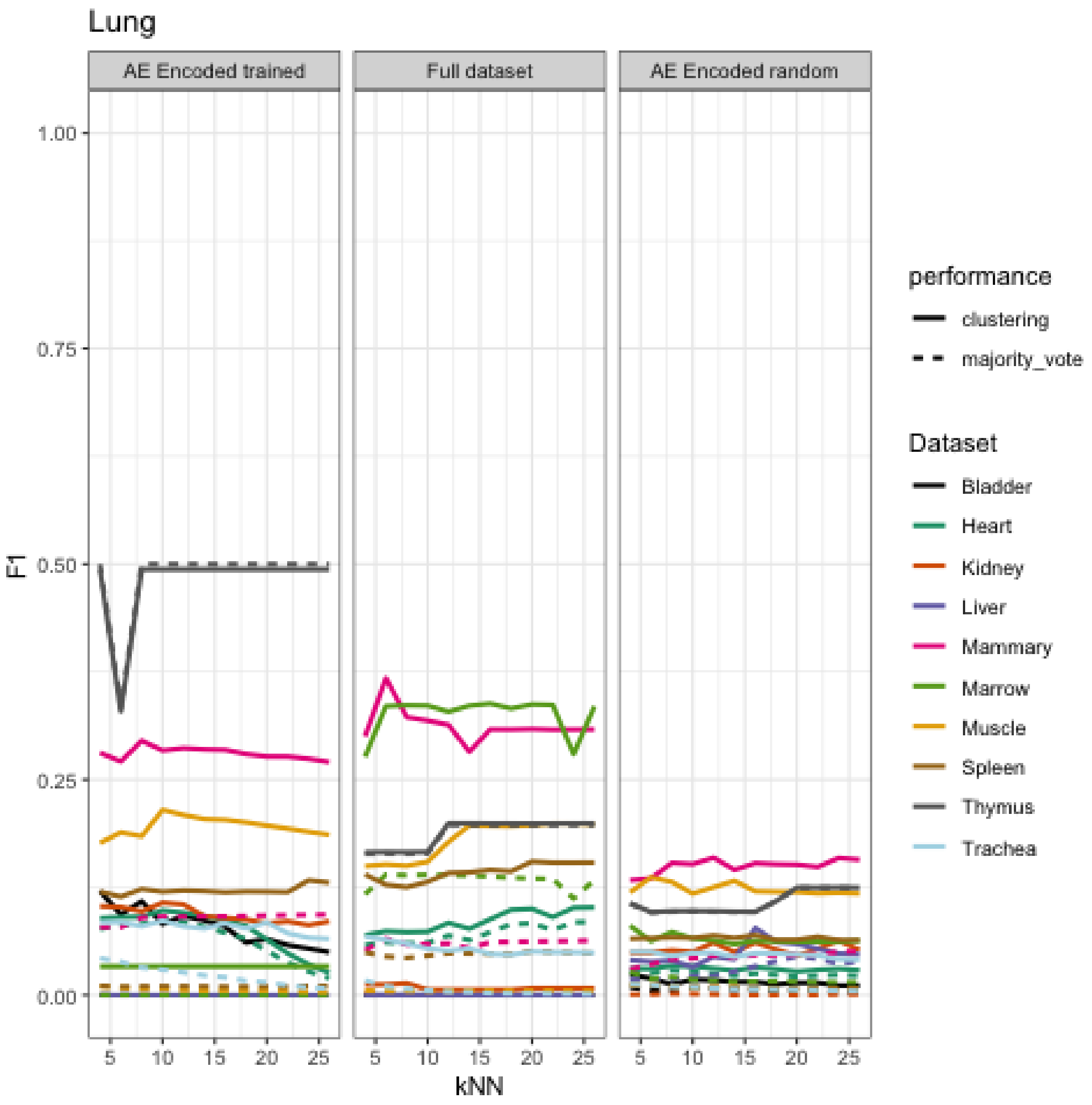

Figure S8: Mean F1 scores for the cell types in the Marrow dataset from Smart-seq2

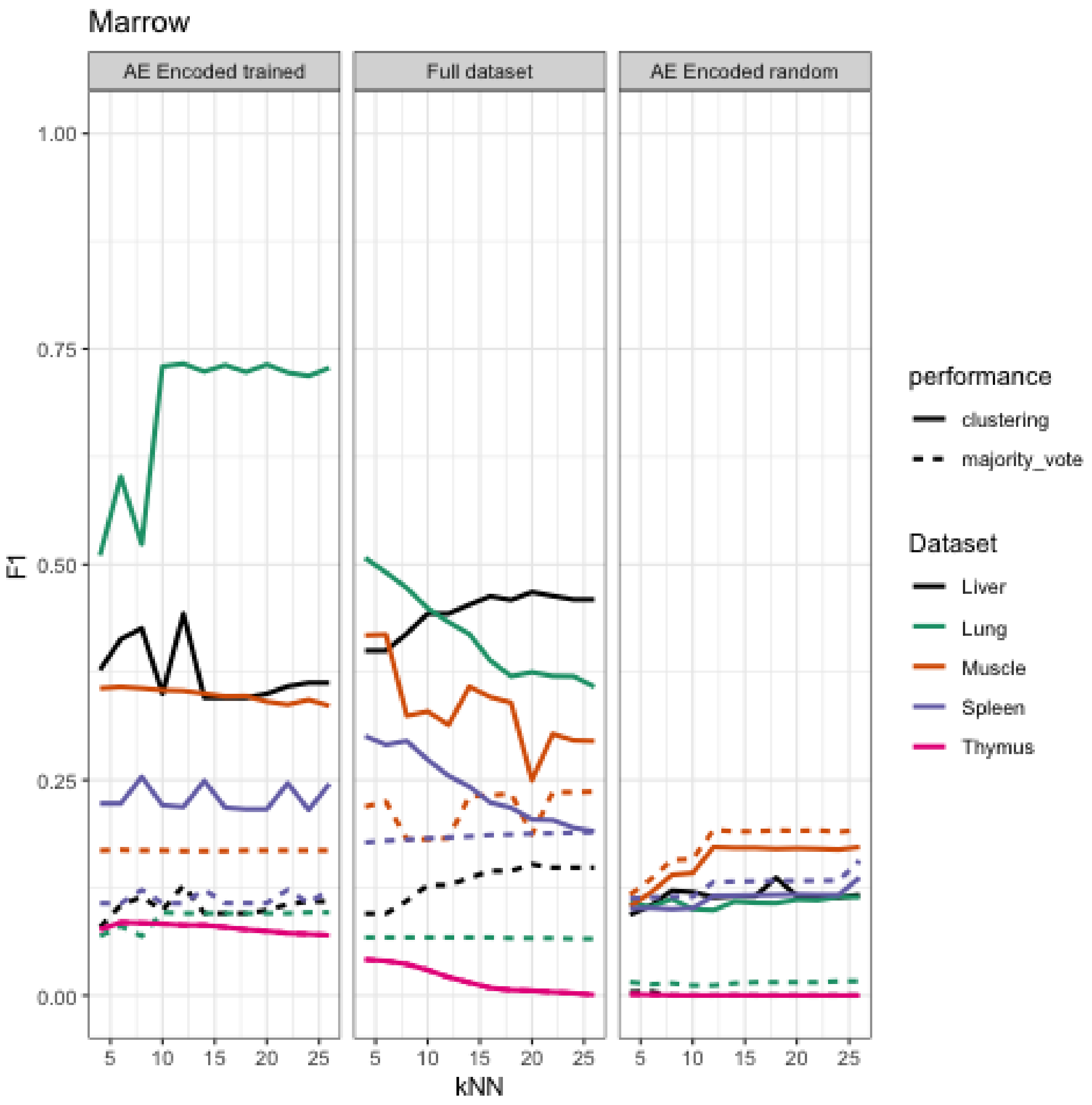

Section H
Gene set enrichment analysis of saliency maps

GSEA was performed on the input using the gradient of the back-propagated saliencies.

Model trained on Muscle dataset from Smart-seq2 without orthogonality constraint, was used to compute the Saliency maps for the each cell type in the Mammary dataset.

Figure S9: Heatmap is showing frequency that each pathway was significant. The Saliency map from each bottleneck layer was thereby used as an input to the GSEA analysis to compute the amount of times a given pathway was significant. The x-axis represents the (nth-bottleneck layer) and the color of the meatmap displays how many times the given Hallmark pathway was found significant, considering that the mammary dataset has 4 cell types. Only hidden units with at least one significant pathway are displayed

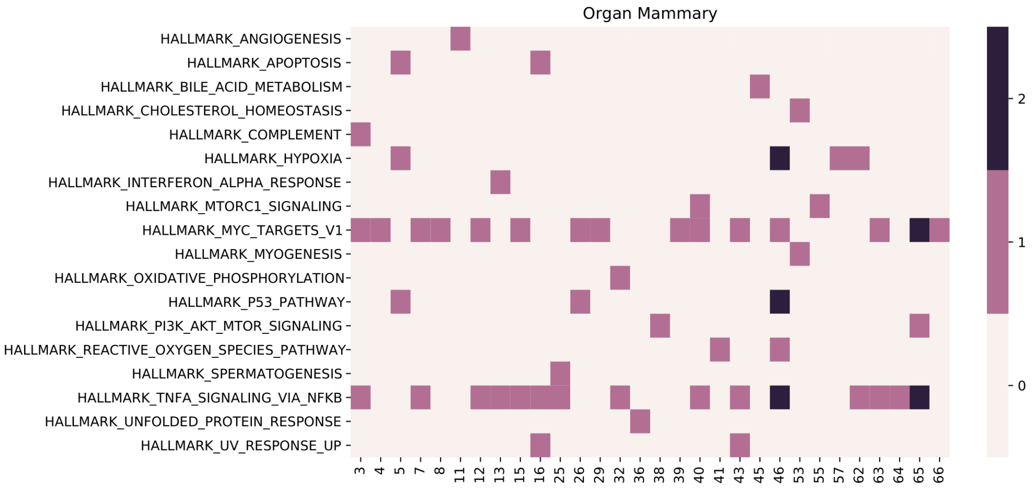

Section I
The autoencoders ability to project Human data

To investigate the autoencoders ability to project data from other species than *mus musculus*. We tested scRNA-seq data from *homo sapiens* in the trained mouse models using 10Xgenomics data as both train and test. The human data is available at Human Cell Atlas Data Portal Single-cell transcriptome profiling of an adult human cell atlas of 15 major organs, see [18]. The human data's gene symbols was converted to mouse symbols using the biomaRt package from R. Subsequently we tested the trained autoencoders ability to project the scRNA-seq data from *homo sapiens* in the bottleneck layer. Below is seen the the autoencoder trained on 10Xgenomics from *Mammary_Gland* from mouse projecting the human data from the *Muscle*.

Figure S10: TSNE of the original Human *Muscle* data

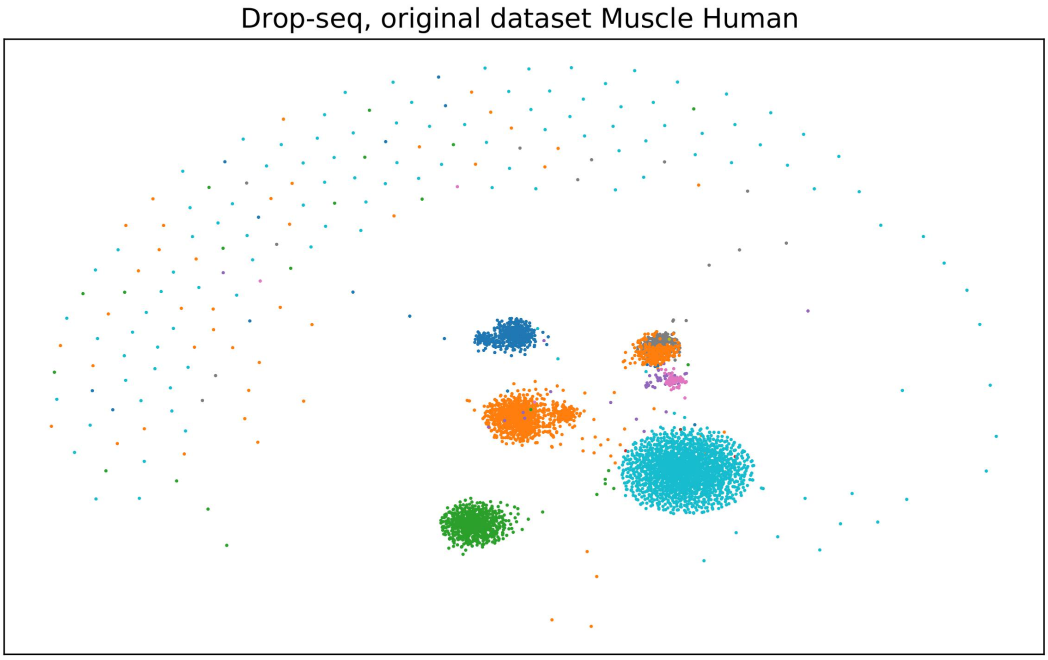

Figure S11: TSNE of the Human *Muscle* data projected on model trained on mouse data from *Mammary_Gland*

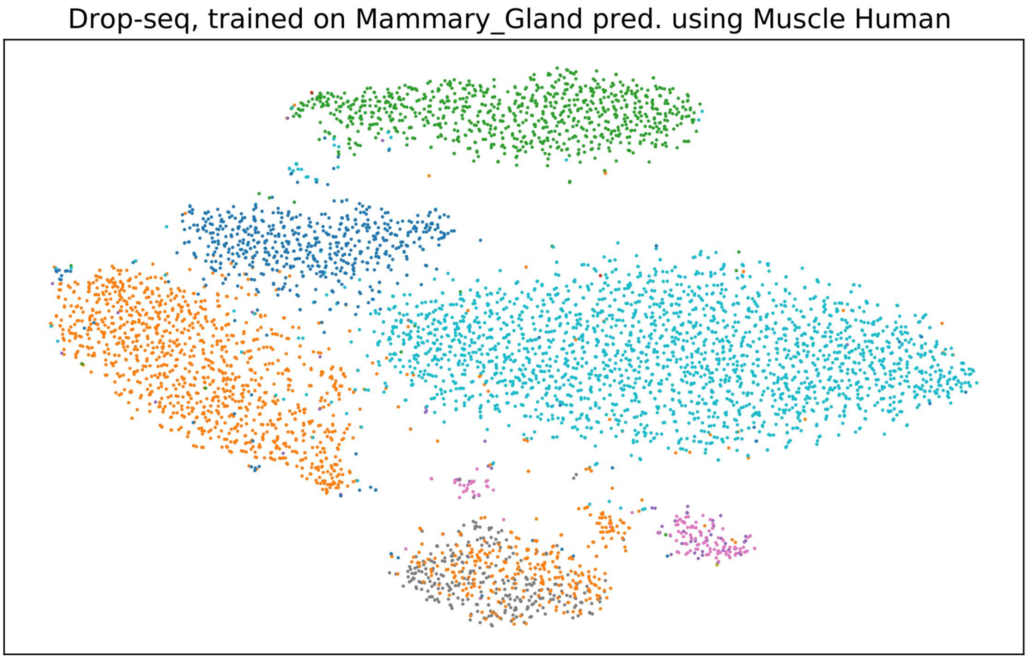

References

[1] G. Eraslan, L. M. Simon, M. Mircea, N. S. Mueller **and** F. J. Theis, "Single-cell rna-seq denoising using a deep count autoencoder," *Nature Communications*, **jourvol** 10, **number** 1, **page** 390, 2019, ISSN: 20411723. DOI: 10.1038/s41467-018-07931-2.

[2] S. Kinalis, F. C. Nielsen, O. Winther **and** F. O. Bagger, "Deconvolution of autoencoders to learn biological regulatory modules from single cell mrna sequencing data," *Bmc Bioinformatics*, **jourvol** 20, **number** 1, **page** 379, 2019, ISSN: 14712105. DOI: 10.1186/s12859-019-2952-9.

[3] N. Bansal, X. Chen **and** Z. Wang, "Can we gain more from orthogonality regularizations in training deep cnns?" *Arxiv*, 2018. [Online]. Available: https://arxiv.org/abs/1810.09102.

[4] T. Stuart, A. Butler, P. Hoffman, C. Hafemeister, E. Papalexi, W. M. Mauck, Y. Hao, M. Stoeckius, P. Smibert **and** R. Satija, "Comprehensive integration of single-cell data," *Cell*, **jourvol** 177, **number** 7, 1888–1902.e21, 2019, ISSN: 10974172, 00928674. DOI: 10.1016/j.cell.2019.05.031.

[5] W. Wang, D. Yang, F. Chen, Y. Pang, S. Huang **and** Y. Ge, "Clustering with orthogonal autoencoder," *Ieee Access*, **jourvol** 7, **number** 99, **pages** 8712494, 62421–62 432, 2019, ISSN: 21693536. DOI: 10.1109/access.2019.2916030.

[6] X. Glorot **and** Y. Bengio, "Understanding the difficulty of training deep feedforward neural networks," *Journal of Machine Learning Research*, **jourvol** 9, **pages** 249–256, 2010, ISSN: 15337928, 15324435.

[7] I. Sutskever, J. Martens, G. Dahl **and** G. Hinton, "On the importance of initialization and momentum in deep learning," 2013.

[8] G. Eraslan, Ĺ. Avsec, J. Gagneur **and** F. J. Theis, "Deep learning: New computational modelling techniques for genomics," *Nature Reviews Genetics*, **jourvol** 20, **number** 7, **pages** 389–403, 2019, ISSN: 14710064, 14710056. DOI: 10.1038/s41576-019-0122-6.

[9] B. Ghojogh **and** M. Crowley, "The theory behind overfitting, cross validation, regularization, bagging, and boosting: Tutorial," *Arxiv*, **page** 23, 2019. [Online]. Available: https://arxiv.org/abs/1905.12787.

[10] V. Perrone, H. Shen, M. Seeger, C. Archambeau **and** R. Jenatton, "Learning search spaces for bayesian optimization: Another view of hyperparameter transfer learning," *Arxiv*, 2019. [Online]. Available: https://arxiv.org/abs/1909.12552.

[11] L. Brocki **and** N. C. Chung, "Concept saliency maps to visualize relevant features in deep generative models," *Arxiv*, 2019. [Online]. Available: https://arxiv.org/abs/1910.13140.

[12] J. T. Springenberg, A. Dosovitskiy, T. Brox **and** M. Riedmiller, "Striving for simplicity: The all convolutional net," *Arxiv*, 2015. [Online]. Available: https://arxiv.org/abs/1412.6806.

[13] A. Liberzon, C. Birger, H. ThorvaldsdĂłttir, M. Ghandi, J. P. Mesirov **and** P. Tamayo, "The molecular signatures database hallmark gene set collection," *Cell Systems*, **jourvol** 1, **number** 6, **pages** 417–425, 2015, ISSN: 24054720, 24054712. DOI: 10.1016/j.cels.2015.12.004.

[14] G. Korotkevich, V. Sukhov **and** A. Sergushichev, "Fast gene set enrichment analysis," *bioRxiv*, 2019. DOI: 10.1101/060012. [Online]. Available: https://www.biorxiv.org/content/early/2019/10/22/060012.

[15] A. Subramanian, P. Tamayo, V. K. Mootha, S. Mukherjee, B. L. Ebert, M. A. Gillette, A. Paulovich, S. L. Pomeroy, T. R. Golub, E. S. Lander **and** J. P. Mesirov, "Gene set enrichment analysis: A knowledge-based approach for interpreting genome-wide expression profiles," *Proceedings of the National Academy of Sciences*, **jourvol** 102, **number** 43, **pages** 15 545–15 550, 2005, ISSN: 0027-8424. DOI: 10.1073/pnas.0506580102. [Online]. Available: https://www.pnas.org/content/102/43/15545.

[16] M. D. Luecken **and** F. J. Theis, "Current best practices in single-cell rna-seq analysis: A tutorial," *Molecular Systems Biology*, **jourvol** 15, **number** 6, 2019, ISSN: 17444292. DOI: 10.15252/msb.20188746.

[17] F. Pedregosa, G. Varoquaux, A. Gramfort, V. Michel, B. Thirion, O. Grisel, M. Blondel, P. Prettenhofer, R. Weiss, V. Dubourg, J. Vanderplas, A. Passos, D. Cournapeau, M. Brucher, M. Perrot **and** E. Duchesnay, "Scikit-learn: Machine learning in Python," *Journal of Machine Learning Research*, **jourvol** 12, **pages** 2825–2830, 2011.

[18] S. He, L.-H. Wang, Y. Liu, Y.-Q. Li, H.-T. Chen, J.-H. Xu, W. Peng, G.-W. Lin, P.-P. Wei, B. Li, X. Xia, D. Wang, J.-X. Bei, X. He **and** Z. Guo, "Single-cell transcriptome profiling of an adult human cell atlas of 15 major organs," *bioRxiv*, 2020. DOI: 10.1101/2020.03.18.996975. eprint: https://www.biorxiv.org/content/early/2020/11/06/2020.03.18.996975.full.pdf. [Online]. Available: https://www.biorxiv.org/content/early/2020/11/06/2020.03.18.996975.
